# Suppression of Rotenone-Treated Human Breast Cancer Stem Cell Survival Using Survivin Inhibitor YM155 is Associated to Oxidative Stress Modulation

**DOI:** 10.31557/APJCP.2020.21.9.2631

**Published:** 2020-09

**Authors:** Resda A Syahrani, Elvira Yunita, Septelia I Wanandi

**Affiliations:** 1 *Molecular Biology and Proteomics Core Facilities, Indonesian Medical Education and Research Institute, Faculty of Medicine, Universitas Indonesia, Jakarta 10430, Indonesia. *; 2 *Master’s Programme in Biomedical Sciences, Faculty of Medicine, Universitas Indonesia, Jakarta 10430, Indonesia. *; 3 *Department of Biochemistry and Molecular Biology, Faculty of Medicine, Universitas Indonesia, Jakarta 10430, Indonesia. *

**Keywords:** Breast cancer stem cells, oxidative stress, surviving, YM155

## Abstract

**Background:**

Despite recent progress in molecular-targeted therapies, breast cancer remains the primary leading cause of cancer related death among women worldwide. Breast cancer stem cells (BCSCs) are believed to be responsible for therapy resistance and cancer recurrence. We recently demonstrated that human BCSCs (CD24-/CD44+) could survive better than their counterpart non-BCSCs (CD24-/CD44-) when treated with rotenone, possibly due to lower levels of reactive oxygen species (ROS) production, high expression of antioxidant manganese superoxide dismutase (MnSOD), and anti-apoptotic survivin. The aim of this study was to verify the role of survivin on human BCSCs survival under oxidative stress modulation by suppressing its expression using YM155, a survivin inhibitor.

**Methods:**

Human BCSCs (ALDH+ cells) were treated with YM155 for 24 h prior to treatment with rotenone for a further 6 h. We determined intracellular superoxide levels were determined using dihydroethidium assay, survivin and MnSOD expression using qRT-PCR, survivin protein level using ELISA, as well as cell viability using trypan blue exclusion and acridine orange/ethidium bromide apoptosis assay.

**Results:**

Suppression of survivin expression using YM155 could reduce the survival of rotenone-treated BCSCs, which may be associated with oxidative stress modulation, as shown by increased ROS levels and decreased MnSOD expression. We confirm that survivin is responsible for maintaining BCSCs survival under oxidative stress modulation. Furthermore, YM155 could modulate oxidative stress in BCSCs by reducing MnSOD expression and increasing ROS levels.

**Conclusion:**

YM155 treatment could be used to overcome BCSCs resistance to oxidative stress-based anticancer therapies.

## Introduction

Breast cancer is one of main cause in cancer mortalities among women worldwide due to a high incidence of recurrence. Conventional breast cancer treatments include surgery, radiotherapy, chemotherapy and hormonal therapy (Ferlay et al., 2015; Nounou et al., 2015). It has been reported (Al-Ejeh et al., 2011; Wicha, 2008) that the recurrence of breast cancer is initiated by a minor population of cancer cells, known as cancer stem cells (CSCs). CSCs have been proven to have characters similar to normal stem cells, such as self-renewal, pluripotency and a high survival rate. Breast CSCs (BCSCs) are heterogeneous and identified based on the presence of several surface antigen markers, such as CD44+, CD24−, and ALDH+. Because BCSCs are considered to be responsible for recurrence, the development of breast cancer therapy should focus on its elimination (Li et al., 2017; Horimoto et al., 2016).

One of the main principle of breast cancer treatment is to treat cancer cells with excessive free radicals. The balance disturbance between free radicals and defense mechanisms of endogenous antioxidant, known as oxidative stress, could lead to cell death (Dayem et al., 2010; Mencalha et al., 2014). We recently demonstrated that human BCSCs (CD24-/CD44+) survived better than their counterpart non-BCSCs (CD24-/CD44-) when treated with rotenone, possibly due to lower levels of reactive oxygen species (ROS) production, high expression of antioxidant MnSOD, and anti-apoptotic survivin (Wanandi et al., 2017).

Survivin is a member of the inhibitor of apoptosis protein (IAP) family (Altieri, 2010). Many studies declared that survivin upregulation correlated with increased relapse and a higher frequency of metastases in breast cancer patients. Moreover, high survivin expression in cancer cells caused resistance to apoptosis induced by various chemotherapeutic agents (Mobahat et al., 2014; Yu et al., 2015). Previous studies have demonstrated (Yamanaka et al., 2011; Cheng et al., 2015) that YM155, the novel small molecule, could suppress survivin expression with minimal effects on the other IAP family member expression levels.

In the present study, we aimed to verify the role of survivin on the survival of human BCSCs under oxidative stress modulation by suppressing its expression using YM155. Additionally, the in vitro antitumor efficacy of YM155 was evaluated via the survival and oxidative stress modulation of rotenone-induced BCSCs.

## Materials and Methods

*Cell culture and morphology*

BCSCs (ALDH+) were isolated from pleural effusion of a patient with metastatic breast cancer provided by Professor Osamu Ohneda (Laboratory of Regenerative Medicine and Stem Cell Biology, Graduate School of Comprehensive Human Sciences, University of Tsukuba, Tsukuba, Japan) and have been established as a cell line, as describe previously (Shiraishi et al., 2017). BCSCs were grown in serum-free DMEM/F12 medium (Gibco^®^, Thermo Fisher Scientific Inc., Waltham, MA, USA) and supplemented with 1% penicillin/streptomycin (Gibco^®^, Thermo Fisher Scientific Inc., Waltham, MA, USA) and 1% amphotericin B (Gibco^®^, Thermo Fisher Scientific Inc., Waltham, MA, USA). The medium was replaced every 2–3 days. The cells were subcultured when the cultures reached 80% to 90% confluence. The standard conditions for cell cultures were 5% CO_2_ and 20% O_2 _at 37^o^C. The morphology of the cells was observed with inverted microscope at 100x magnification (OPTIKA Srl, Ponteranica, Italy).

*YM155 and rotenone treatment*

YM155 (Cayman Chemicals, Ann Arbor, MI, USA) was dissolved in dimethyl sulfoxide (DMSO) (Sigma-Aldrich, Inc. St. Louis, MO, USA) and diluted to 100 nM using serum-free culture medium [0.0009% (v/v) final concentration of DMSO in the culture medium]. Rotenone (Sigma-Aldrich, Inc. St. Louis, MO, USA) was dissolved in DMSO and diluted to 0.5, 5, and 50 µM using a serum-free culture medium [0.2% (v/v) final concentration of DMSO in the culture medium].

A total of 1×10^5^ cells per well in 12-well plates were treated with 100 nM YM155, while the control was treated with 0.0009% DMSO for 24 h. Cells were then treated with either rotenone at various concentrations or with 0.2% DMSO (control). After 6 h of incubation, the cells were harvested for further analysis. Cell viability was determined using the trypan blue exclusion assay.

*In vitro assay for ROS detection*

Intracellular ROS production was carried out using a superoxide sensitive probe dihydroethidium (DHE) assay (Invitrogen™ Molecular Probes™, Thermo Fisher Scientific Inc., Waltham, MA, USA), as described previously (Hardiany et al., 2017). Briefly, 2 × 10^4^ cells were collected and twice washed with sterile phosphate buffer saline (PBS) (Gibco^®^, Thermo Fisher Scientific Inc., Waltham, MA, USA). Cells were suspended in 500 µL of PBS and loaded with 20 µM DHE dye for 30 min at 37°C in the dark. Fluorescence intensity was measured immediately using a fluorimeter (Varioskan™ Flash Multimode Reader, Thermo Fisher Scientific, Inc. Waltham, MA, USA) with excitation at 480 nm and emission at 585 nm.

*Total RNA preparation and real-time RT-PCR*

Total RNA was isolated from cells using Tripure Isolation Reagent (Roche Applied Science, Basel, Switzerland) according to the manufacturer’s instruction. Total RNA concentration was quantified using a spectrophotometer at 260 nm (Varioskan™ Flash Multimode Reader, Thermo Fisher Scientific, Inc. Waltham, MA, USA). Quantitative real-time PCR was performed using KAPA SYBR^®^ FAST One-step qRT-PCR (Kapa Biosystems, Wilmington, Massachusetts, USA) in an Exicycler™ 96 (Bioneer, Daejeon, Republic of Korea) according to the manufacturer’s instruction. Primers used for 18S rRNA expression were AAACGGCTACCACATCCAAG (forward) and CCTCCAATGGATCCTCGTTA (reverse), for survivin expression were GCCAGATGACGACCCCATAGAGGA (forward) and TCGATGGCACGGCGCACTTT (reverse), whereas for MnSOD expression were GCACTAGCAGCATGTTGAGC (forward) and ACTTCTCCTCGGTGACGTTC (reverse). Annealing temperature for both 18S rRNA and MnSOD primers were 60ºC, meanwhile for survivin primers was 61ºC. The qRT-PCR was performed as previously described (Wanandi et al., 2017; Hardiany et al., 2017). All reactions were performed in triplicate and relative mRNA expression levels were calculated using Livak formula.

*Determination of surviving protein level*

Total protein was isolated from human BCSCs using RIPA lysis buffer (Invitrogen™, Thermo Fisher Scientific Inc., Waltham, MA, USA)) according to the manufacturer’s instructions. Concentration of total protein was measured by plotting to the Bovine Serum Albumin (BSA) (Sigma-Aldrich, Inc. St. Louis, MO, USA) standard curve using spectrophotometer (Varioskan™ Flash Multimode Reader, Thermo Fisher Scientific, Inc. Waltham, MA, USA) at λ 280nm. A total of 20 µL of protein lysate was used to measure survivin protein using PathScan Total Survivin Sandwich ELISA Kit (Cell Signaling Technology, Danvers, MA, USA), as per the manufacturer’s protocol. The absorbance was measured using a spectrophotometer at 450 nm (Varioskan™ Flash Multimode Reader, Thermo Fisher Scientific, Inc. Waltham, MA, USA)). The absorbance was divided by the total protein concentration.

*Measurement of MnSOD specific activity*

MnSOD enzyme activity was analysed by using Randox Superoxide Dismutase Kit (Randox Laboratories Ltd., County Antrim, UK), as previously described (Hardiany et al., 2017). Briefly, inhibition of Cu/ZnSOD was performed by adding sodium cyanide 5 mM into each sample. The absorbance was measured using spectrophotometry at λ 505nm (Varioskan™ Flash Multimode Reader, Thermo Fisher Scientific, Inc. Waltham, MA, USA) and the percentage of inhibition was plotted to the standard curve to obtain the enzyme activity calculation. Specific activity of MnSOD enzyme represents enzyme activity (in units) per mg total protein.

*Determination of apoptosis using acridine orange/ethidium bromide (AO/EB)*

The dye mix for the AO/EB staining was 100 µg/mL acridine orange and 100 µg/mL ethidium bromide in PBS (Ribble et al., 2005). A volume of 2 μL of AO/EB solution was added to 25 µL of cell suspension and mixed gently. The mixture was incubated in the dark for 3 min. Next, 10 μL of the mixture was placed onto a microscope slide, covered with a glass coverslip, and at least 100 cells were examined using a fluorescence microscope (Olympus Corporation, Tokyo, Japan).

*Statistical analysis*

All values obtained were compared with those of the control cells and presented as mean ± standard deviation. Statistical evaluation of significant differences was performed using Student’s t test.

## Results

*Optimization of YM155 treatment*

**Figure 1:**
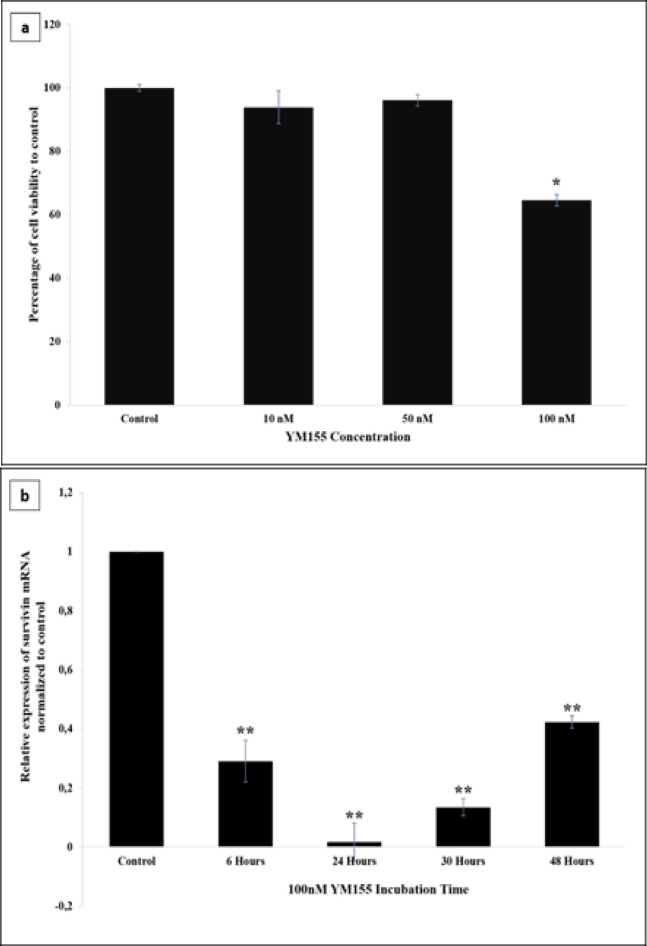
Optimization of BCSCs Treatment with YM155. BCSCs were seeded at a density of 1×10^5^ cells per well in 12-well plates and incubated overnight. Cells were then treated with YM155 at various concentrations for a range of incubation times. All experiments were performed in triplicate. *p<0.05 vs. control; **p<0.01 vs. control. (a) Cell viability was calculated as a percentage of viable cells treated with various concentrations of YM155 compared to DMSO treatment as control. (b) Relative mRNA expression of survivin in YM155-treated BCSCs was normalized to that of DMSO-treated cells (control)

To verify the role of YM155 in inhibiting survivin, we first optimized YM155 treatment by treating BCSCs with various concentrations and various incubation times. Optimization of the concentration was carried out using 10, 50, and 100 nM YM155 for 24 h. The results showed that 100 nM YM155 significantly decreased cell viability by ~36% compared with control cells treated with DMSO, as shown in Figure 1a. We also optimized the incubation time using 100 nM YM155 by treating BCSCs for 6, 24, 30, and 48 h, respectively. Figure 1b demonstrated that although all incubation times of YM155 significantly decreased survivin expression in BCSCs, the optimal incubation time was 24 h which decreased the expression by ~98% compared to control. 

*Effect of rotenone on surviving expression of BCSCs after YM155 treatment*

**Figure 2:**
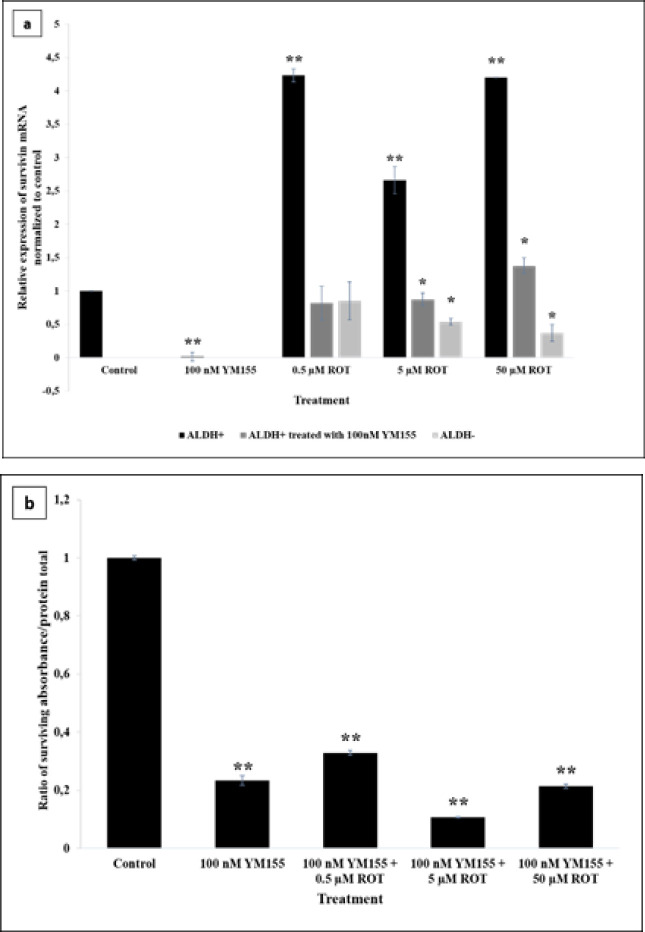
Effect of YM155 Treatment on Survivin of BCSCs after YM155 Treatment. BCSCs were firstly treated with 100 nM YM155 for 24 h, followed by 6 h incubation with various concentration of rotenone. Experiments were performed in triplicate. *p<0.05 vs. control; **p<0.01 vs. control. (a) Relative mRNA expression of survivin in rotenone-induced BCSCs treated with 100 nM YM155 was calculated by normalizing to DMSO-treated cells (control). (b) Level of survivin protein in rotenone-induced BCSCs treated with 100 nM YM155 was calculated as a ratio of the absorbance of total survivin divided by the total protein concentration compared to DMSO treatment as control

Modulation of oxidative stress in BCSCs and non-BCSCs was achieved using rotenone, a complex I electron transport chain inhibitor, in order to simulate chemoradiation therapy based on ROS generation, particularly superoxide radicals. Rotenone was used at concentrations of 0.5, 5, and 50 μM, respectively, based on our previous study (Wanandi et al., 2017). In Figure 2a, we showed that, following all concentration of rotenone treatment, expression levels of survivin mRNA in BCSCs (ALDH+ cells) was significantly higher than that in control. In contrast to that data, survivin expression in the non-BCSCs (ALDH- cells) were decreased after 5 and 50 μM rotenone treatment. After YM155 treatment, all concentrations of rotenone could increase the survivin mRNA expression, nevertheless, the increase was not significant compared to BCSCs with rotenone only. Furthermore, Figure 2b demonstrated that YM155 also significantly suppressed the survivin protein in BCSCs either with or without rotenone.

*Effect of rotenone on the viability of BCSCs after YM155 treatment*

**Figure 3:**
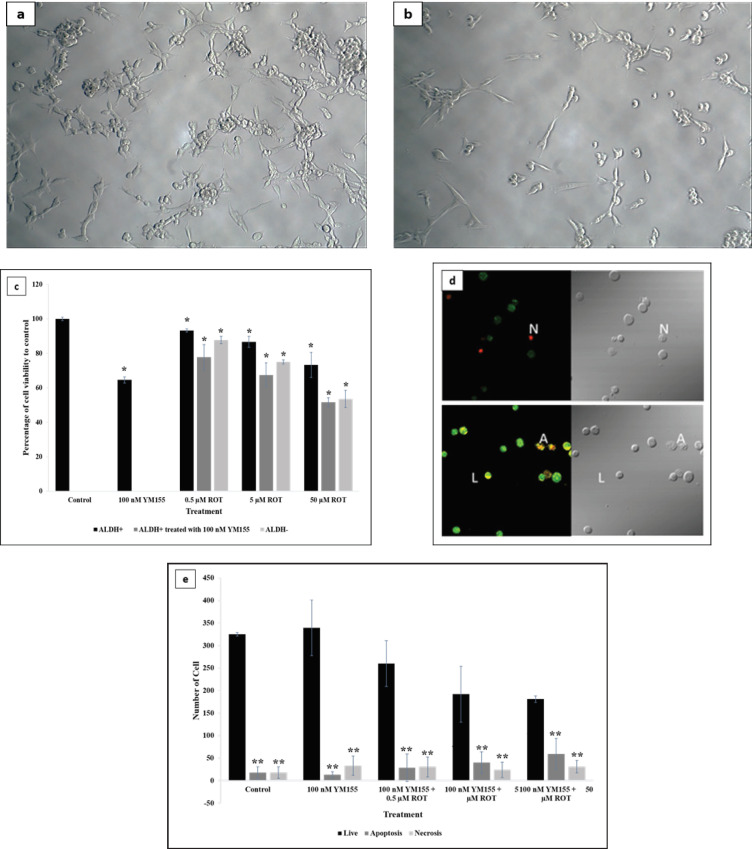
Effect of Rotenone on Morphology, Viability, and Apoptosis of BCSCs after YM155 Treatment. *p<0.05 vs. control; **p<0.01 vs. respective live cell. (a) Morphology of BCSCs treated with DMSO as control, observed using inverted microscope at 100x magnification. (b) Morphology of BCSCs treated with YM155 and rotenone, observed using inverted microscope at 100x magnification. BCSCs was treated with 100 nM YM155 for 24 h followed by 50 μM rotenone for 6 h. (c) Cell viability was calculated as a percentage of rotenone-induced BCSCs treated with 100 nM YM155 compared to DMSO treatment as control. (d) Viable cells show a normal green nucleus, early apoptotic cells have bright green nucleus with condensed or fragmented chromatin, late apoptotic cells display condensed and fragmented orange chromatin, and cells that have died from direct necrosis have a structurally normal orange nucleus [17]. (e) Number of live, apoptotic, and necrotic cells analyzed by AO/EB staining

Under microscopic observation, Figure 3a showed that the morphology of BCSCs was tend to stick together and form mammospheres (Wanandi et al., 2017). After YM155 and rotenone treatment, there was not any differences in morphology, as shown in Figure 3b. A comparative analysis of viability between BCSCs and non-BCSCs was performed based on the percentage of rotenone-treated to control (DMSO-treated) cells. In Figure 3c, we revealed that rotenone treatment could significantly decrease cell viability in both BCSCs and non-BCSCs. Nevertheless, the viability of BCSCs was higher than the non-BCSCs, particularly when treated with 50 µM rotenone. We then evaluated the effect of YM155 on cell viability. The viability of YM155-treated BCSCs was significantly reduced following rotenone treatment in a concentration-dependent manner compared with their counterpart without YM155 treatment and was even lower than the non-BCSCs. We further analyzed the effects of rotenone on apoptosis and necrosis of YM155-treated BCSCs using AO/EB staining and visualized using a fluorescence microscope, as shown in Figure 3d. Viable cells showed a normal green nucleus, early apoptotic cells showed a bright green nucleus with condensed or fragmented chromatin, late apoptotic cells displayed condensed and fragmented orange chromatin, and cells that have died from direct necrosis have a structurally normal orange nucleus (Ribble et al., 2005). Figure 3e showed various levels of apoptosis and necrosis with the different treatments. Our results demonstrated that, following rotenone treatment, the number of BCSCs underwent apoptosis was gradually increased in concentration-dependent manner in line with the decrease of viable cells. We also observed insignificant increase of cell necrosis that could affect the viable cell count.

*Effect of rotenone on oxidative stress of BCSCs after YM155 treatment*

**Figure 4:**
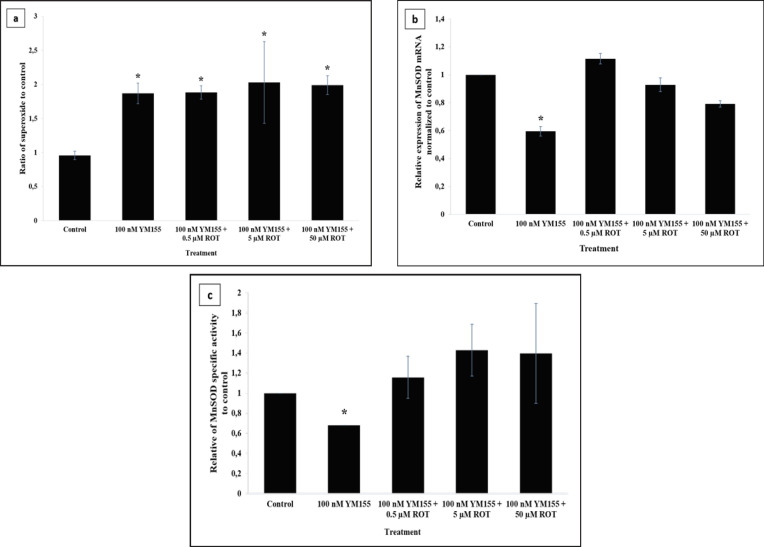
Effect of Rotenone on Superoxide Anion, MnSOD Expression, and MnSOD Specific Activity Levels of BCSCs after YM155 Treatment. *p<0.05 vs. control. (a) Superoxide anions level were assessed by DHE assay. Superoxide radical formation was calculated as a ratio of rotenone-induced BCSCs treated with 100 nM YM155 compared to DMSO-treated cells (control). (b) Relative mRNA expression of MnSOD in YM155-treated BCSCs was normalized to that of DMSO treatment as control. (c) Specific activity of MnSOD was calculated as a ratio of rotenone-induced BCSCs treated with 100 nM YM155 compared to that of DMSO-treated cells (control)

To determine oxidative stress, we investigated the superoxide levels, MnSOD mRNA expression and activity level. The effect of rotenone on superoxide levels in BCSC was determined by the DHE intensity in rotenone-treated cells normalized to DMSO-treated cells as a control. All treatment, with or without rotenone, significantly increased superoxide anion levels in BCSCs higher than control as shown in Figure 4a. As YM155 may involve in superoxide production, we also analyzed the expression and activity of MnSOD antioxidant, in order to evaluate the adaptive response of BCSCs. MnSOD mRNA expression levels were measured in BCSCs based on relative expression normalized to the control. Surprisingly, we found that following treatment with YM155 alone, BCSCs showed a significant decrease in MnSOD mRNA expression levels compared with that in the control, as demonstrated in Figure 4b. Furthermore, Figure 4c revealed that there was also a decrease in the specific activity compared with the control. However, rotenone exposure of all concentrations recovered both the MnSOD mRNA expression and specific activity into the levels similar to the control.

## Discussion

The success of cancer therapy is determined by its ability to affect cancer cells survival. When cancer cells are sensitive to therapy, their survival will decrease and can lead to elimination of the cancer cells. Unfortunately, conventional principle of anticancer therapy is generally based on the assumption that all cancer cells have similar potential for malignancy, without considering the presence of BCSCs (Li et al., 2017; Horimoto et al., 2016). Previous studies (Al-Ejeh et al., 2011; Wicha, 2008) described CSCs as a minor population of breast cancer cells that has roles in high survival rates and resistance to therapies. Therefore, the development of therapy targeted to decrease BCSCs survival is urgently required to eliminate cancer at the root. 

It should be noted that most chemo- and radiotherapy acts by modulating oxidative stress in cancer cells. In the present study, rotenone was used to simulate the effect of free radical-based anticancer therapies. Rotenone blocks electron flow from NADH to coenzyme Q at complex I of the respiratory chain in mitochondria and increases superoxide radicals, leading to the induction of cytotoxicity (Goncalves et al., 2011). As demonstrated in our previous study (Wanandi et al., 2017), we noticed that BCSCs were more viable than non-BCSCs when treated with increasing concentrations of rotenone. Combined with the results of survivin mRNA expression, substantially higher expression of survivin in rotenone-treated BCSCs is thought to precede their higher viability compared with those in rotenone-treated non-BCSCs. Thus, we presumed that the increase of survivin mRNA expression could be another mechanism employed by BCSCs to survive oxidative stress modulation.

Furthermore, we evaluated the potential of survivin as an ideal target for breast cancer treatment. Survivin is a protein that facilitates inhibition of cancer apoptosis (Chen et al., 2016). Considering that survivin is overexpressed in cancer and expressed at low levels (or absent) in most normal tissues, this suggests that survivin expression dysregulation may generate an ability to induce apoptosis. Many studies have focused on developing strategies to target survivin for cancer therapies. Developing drugs that use survivin as a target might initially seem difficult because survivin is neither an enzyme nor a cell surface protein. However, considerable progress has been made to obtain optimal efficiency in survivin suppression (Altieri, 2008). Recent studies have indicated that YM155 could suppress survivin expression by directly binding to its promoter (Yamanaka et al., 2011; Cheng et al., 2015). The present study confirmed that YM155 decreased survivin mRNA expression and protein levels.

In this study, suppression of survivin seems to have an impact on viability, as shown by decreasing of BCSCs viability. The reduction of cell viability may be due to apoptosis rather than necrosis. Survivin has been reported as an inhibitor for caspase that induces the negative regulation of apoptosis (Chen et al., 2016; Altieri, 2008). The present study confirmed that by inhibiting survivin with YM155 would lead to enhancement of apoptosis regulation.

Interestingly, we found that YM155 could also increase superoxide production, which has not been reported previously. As YM155 molecule contains ubiquinone (Tao et al., 2012), we suggest that the increased ROS production may be due to the conversion of ubiquinone into semi-quinone. Unexpectedly, the present study reveals that MnSOD expression in YM155-treated BCSCs was suppressed, both at the mRNA and specific activity levels. The mechanism of MnSOD inhibition remains to be elucidated. In addition to our previous study that demonstrated the increase of MnSOD expression and activity in BCSCs after rotenone exposure (Wanandi et al., 2017), we here indicated that, after YM155 treatment, rotenone can also increase the MnSOD expression and activity of BCSCs even though there is not significant differences compared to control. However, the present study reports for the first time that YM155 could modulate oxidative stress in BCSCs by suppressing MnSOD expression and increasing ROS levels.

In conclusion, survivin plays an important role in BCSCs survival by modulating oxidative stress. We propose that YM155 may offer a novel therapeutic option for the eradication of BCSCs and overcome BCSCs resistance in oxidative stress-based anticancer therapies.
